# AI-Enhanced evaluation of YouTube content on post-surgical incontinence following pelvic cancer treatment

**DOI:** 10.1016/j.ssmph.2024.101677

**Published:** 2024-05-04

**Authors:** Alvaro Manuel Rodriguez-Rodriguez, Marta De la Fuente-Costa, Mario Escalera-de la Riva, Borja Perez-Dominguez, Gustavo Paseiro-Ares, Jose Casaña, Maria Blanco-Diaz

**Affiliations:** a Physiotherapy and Translational Research Group (FINTRA-RG), Institute of Health Research of the Principality of Asturias (ISPA), University of Oviedo, 33011, Oviedo, Spain; b Faculty of Medicine and Health Sciences, University of Oviedo, 33006, Oviedo, Spain; c Exercise Intervention for Health Research Group (EXINH-RG), Department of Physiotherapy, University of Valencia, 46010, Valencia, Spain; d Psychosocial Intervention and Functional Rehabilitation Research Group, Faculty of Physiotherapy, University of A Coruña, 15006, Coruña, Spain

**Keywords:** Cancer, Incontinence, Surgery, PCA, t-SNE, UMAP, Information, Quality, Youtube, HeatMap, Dendrogram, DISCERN, GQS, JAMA, PEMAT, MQ-VET

## Abstract

**Background:**

Several pelvic area cancers exhibit high incidence rates, and their surgical treatment can result in adverse effects such as urinary and fecal incontinence, significantly impacting patients' quality of life. Post-surgery incontinence is a significant concern, with prevalence rates ranging from 25 to 45% for urinary incontinence and 9–68% for fecal incontinence. Cancer survivors are increasingly turning to YouTube as a platform to connect with others, yet caution is warranted as misinformation is prevalent.

**Objective:**

This study aims to evaluate the information quality in YouTube videos about post-surgical incontinence after pelvic area cancer surgery.

**Methods:**

A YouTube search for "*Incontinence after cancer surgery*" yielded 108 videos, which were subsequently analyzed. To evaluate these videos, several quality assessment tools were utilized, including DISCERN, GQS, JAMA, PEMAT, and MQ-VET. Statistical analyses, such as descriptive statistics and intercorrelation tests, were employed to assess various video attributes, including characteristics, popularity, educational value, quality, and reliability. Also, artificial intelligence techniques like PCA, t-SNE, and UMAP were used for data analysis. HeatMap and Hierarchical Clustering Dendrogram techniques validated the Machine Learning results.

**Results:**

The quality scales presented a high level of correlation one with each other (*p* < 0.01) and the Artificial Intelligence-based techniques presented clear clustering representations of the dataset samples, which were reinforced by the Heat Map and Hierarchical Clustering Dendrogram.

**Conclusions:**

YouTube videos on "*Incontinence after Cancer Surgery*" present a "*High*" quality across multiple scales. The use of AI tools, like PCA, t-SNE, and UMAP, is highlighted for clustering large health datasets, improving data visualization, pattern recognition, and complex healthcare analysis.

## Highlights

• YouTube videos on incontinence after cancer surgery were rated as high-quality across multiple assessment tools.
• Significant correlations were found between 5 quality scales (DISCERN, GQS, JAMA, PEMAT, MQ-VET).
• AI techniques like PCA, t-SNE and UMAP enabled distinct sample clustering without human bias.
• Heatmaps and dendrograms validated graphically the AI analysis, differentiating high and low quality videos.
• Academic, health institutions and professionals had the highest quality videos; non-health individuals the lowest.

## 1 Introduction

Prostate and colorectal cancers are the second and third most common cancers in men, after lung cancer, according to the International Agency for Research on Cancer, part of the World Health Organization (WHO)(International Agency for Research on Cancer, 2020). Colorectal cancers (including colon, rectum, anus, etc.) are the second most common cancers in women after breast cancer, per the same source(Sung et al., 2021). While advances in radiation, chemotherapy, immunotherapy, and targeted therapies have expanded treatment options for many cancers, surgical resection remains the primary treatment for malignancies of the bladder, prostate, cervix, uterus, rectum, and anus (Pallan et al., 2021). Pelvic cancer surgeries like radical prostatectomy, cystectomy, hysterectomy, and abdominoperineal resection require extensive training and skill to balance cancer control with quality of life considerations such as continence and sexual function. Improvements in minimally invasive and robotic-assisted surgical platforms are enhancing access and precision for pelvic cancer resections while aiming to reduce collateral damage and morbidity. However, surgical quality and experience remain paramount for optimizing outcomes in pelvic oncology(Tevis & Kennedy, 2016).

This study recognizes the urgent need for rigorous evaluation of health information quality, especially given the far-reaching implications of misinformation on patient outcomes. This research not only seeks to bridge this gap but also to underscore the critical role of advanced analytical techniques in discerning the quality of health education provided to the public.

Incontinence is a potential side effect of cancer surgeries, particularly for the aforementioned cancer types(Nocera et al., 2021). Incontinence after rectal or prostate cancer treatment refers to the loss of urinary or bowel control(Cascales-Campos et al., 2021), leading to unintentional leakage or inability to regulate urine or feces (Bardsley, 2016; Ng et al., 2015). Incontinence is also a distressingly common complication that profoundly impacts the quality of life (QoL) for many cancer survivors after undergoing invasive pelvic surgeries(Gonçalves, 2010). However, despite significant advancements in surgical techniques over the past decades, postoperative incontinence remains a challenging problem that affects patients both physically and psychologically. Reported prevalence varies widely based on cancer type, surgical approach, and how outcomes are measured, with urinary incontinence rates ranging from 5% to 70% with most studies reporting 25–45% and fecal incontinence between 9% and 68%(Wu et al., 2014), increasing prevalence with age (Pasricha & Staller, 2021).

The loss of urinary and bowel control can profoundly affect cancer survivors emotionally and practically, potentially causing social isolation, sexual intimacy issues, embarrassment, reduced self-esteem, and decreased overall QoL (Pizzol et al., 2021; Sazonova et al., 2022). Constant leakage and involuntary loss of control over basic bodily functions presents unique psychological and sexual challenges for survivors as they cope with the impact on their well-being and sexuality, among other areas(Frigerio et al., 2022; Pičmanová & Procházka, 2021).

In recent years, the advent of social media has transformed how individuals seek and share health-related information. Cancer surgery survivors are no exception, as they increasingly turn to e-health(Åström et al., 2021) and social networks (Howard-Jones et al., 2022). YouTube attracts 95% of internet users worldwide, making it the largest video-sharing platform globally. With over 122 million daily active users and 1 billion hours of content viewed per day, YouTube has a massive user base that cements its position as the most prominent video website(Ceci, 2023; Omnicore, 2021). YouTube's extensive reach and use is evident, rendering it one of the most prominent platforms for patients to access health information(Rodriguez-Rodriguez, Blanco-Diaz, Lopez-Diaz, de la Fuente-Costa, Sousa-Fraguas, et al., 2021). As the most utilized social network and biggest video site worldwide, YouTube offers a vast repository of user-generated content related to cancer surgery and its aftermath(Di Bello et al., 2022; Salman & Bayar, 2021).

YouTube's accessibility and user-friendly interface make it a preferred destination for cancer surgery survivors seeking information about their specific pathologies, treatment options, potential side effects, and strategies for coping with postoperative challenges(Bahar-Ozdemir et al., 2022). With a simple search, survivors can access numerous videos uploaded by healthcare professionals, patient advocates, fellow survivors, and others, sharing experiences and insights. YouTube's visual and audio formats allow survivors to connect emotionally with the content, gaining a deeper understanding of the challenges and triumphs faced by others who have undergone similar procedures(Brar et al., 2021; Sylvia Chou et al., 2011). Engaging in online discussions, joining patient support groups, and participating in virtual events on YouTube enables cancer surgery survivors to find a sense of community and support that may not be easily accessible in their offline lives(Cakmak & Mantoglu, 2021). By interacting with others through comments and messages, survivors can seek advice, share their own experiences, and learn from a diverse range of perspectives. The platform fosters a culture of knowledge exchange and empowerment, where cancer survivors can find validation, encouragement, and solidarity during their post-treatment recovery(Özkent & Kılınç, 2023).

However, while YouTube offers valuable health-related content, it is important for cancer surgery survivors to exercise caution and critical thinking when navigating the platform. The vastness of YouTube's content also means that misinformation and unsubstantiated claims can circulate freely, potentially leading to confusion and misinformation(Sui et al., 2022). Healthcare professionals play a vital role in guiding cancer survivors to credible YouTube channels and encouraging them to verify information with reputable medical sources(Pratsinis et al., 2021). By leveraging the potential of YouTube while promoting evidence-based practices, healthcare providers can enhance their patients' digital health literacy and empower them to make well-informed decisions about their cancer care journey(Baran & Yilmaz Baran, 2021; Madathil et al., 2015).

Furthermore, the application of Artificial Intelligence (AI) in analyzing health information quality represents a novel approach that significantly enhances pattern recognition and data stratification. The incorporation of AI techniques such as PCA, t-SNE, and UMAP is instrumental in managing the complexity and volume of data on platforms like YouTube, enabling a comprehensive and nuanced assessment of content quality.

As the internet has become a common source of health information for many patients, concerns exist regarding the variable quality of uncontrolled online content from platforms like YouTube. Studies show that most patients access online health information independently without input from healthcare professionals. While the internet can help to fill knowledge gaps, patients often lack the necessary skills to appraise the sources' credibility. The primary aim of this research is to examine the quality and accuracy of health information that patients access through online media-sharing platforms, like YouTube, about a pathology with a high prevalence both in men and women. The secondary objective is to use the insights gained from the analysis to inform healthcare providers and patients about reliable and trustworthy sources of online health information thereby enhancing e-health engagement and education. To achieve these objectives, the study utilizes five validated quality assessment tools (such as DISCERN, GQS, JAMA, PEMAT and MQ-VET) and artificial intelligence techniques (such as PCA, t-SNE and UMAP), the study analyzes a sample of publicly available YouTube videos on this health topic to evaluate their educational value and reliability.

## 2 Methods

In this analytical cross-sectional study, it is sought to evaluate the quality and reliability of YouTube videos pertaining to post-surgical incontinence after pelvic cancer surgery. Inspired by the methodological frameworks proposed by Muhammed et al. (Muhammed & Merve, 2023), Shah et al. (Shah et al., 2022) and Coşkun et al. (Coşkun et al., 2023), our approach utilized a combination of validated quality assessment tools and advanced artificial intelligence techniques to perform a comprehensive analysis of video content. Emulating and adapting these methodologies, our study employed a combination of five validated quality assessment instruments—namely DISCERN, GQS, JAMA, PEMAT, and MQ-VET—to rigorously evaluate the content of selected YouTube videos. To delve deeper into the data structure and uncover patterns that may not be immediately apparent through traditional analysis, some artificial intelligence techniques such as PCA, t-SNE, and UMAP were incorporated. This innovative blend of evaluative tools and AI-driven analysis was aimed at capturing a multi-dimensional perspective of the educational value and reliability of the video content, thereby providing a comprehensive insight into its potential impact on patients' understanding and management of their health conditions post-surgery. The selection criteria for videos, data extraction procedures, and analytic techniques were meticulously designed to ensure the replicability of the study and the validity of its findings, with the overarching goal of contributing to the enhancement of digital health literacy and patient education in the context of e-health platforms.

### 2.1 Recruitment

**Fig. 1:**
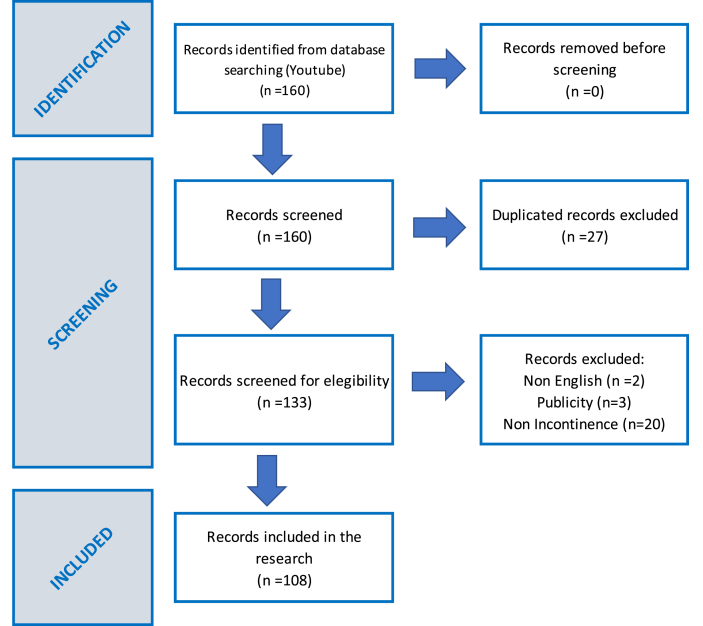
PRISMA flow diagram of records included in the research.

A search on http://www.youtube.com was conducted on March 22nd, 2023, using the search terms *'Incontinence after cancer surgery'*. A total of 160 videos were selected from the available options, without using any filters, with the aim of replicating a simple search strategy that any person could perform. YouTube sorted the video results based on their relevance using their patented ranking algorithm on that specific day. All the videos' links were then added to a spreadsheet and screened for duplicates. The research team applied the exclusion criteria, removing videos in non-English language, duplicated videos, advertisements-related videos and videos unrelated to incontinence. Finally, 108 videos were assigned to two different examiners with expertise in the field who independently viewed, analyzed, and evaluated them over a three-week period, as can be seen in the flow diagram that followed the PRISMA recommendations (Moher et al.; Haddaway et al., 2022) (see Fig. 1).

### 2.2 Statistical analysis

#### 2.2.1 Outcome measures

The descriptive characteristics of each video were collected, including upload date, days online, length (duration in minutes and seconds), number of views, likes, comments, and subscribers. Based on geographical location, videos were classified by continent of origin: Africa, Americas, Asia, Australia, Europe. Videos were categorized into six groups according to author/source: Academic Institutions, Media (newspapers, TV, etc.), Health Institutions, Non-Governmental Organizations (NGOs), Healthcare Individuals (healthcare workers, etc.), and Non-Healthcare Individuals (patients sharing their experiences in this matter).

Additionally, the target audience gender was also noted (Male, Female, Both), along with whether the video mentioned chemotherapy (Yes/No). The type of incontinence was recorded (Urinary, Fecal, Both) as well as the cancer type (Prostate, Uterus, Bladder, Colorectal, Other). Finally, the mentioned intervention type in the video was documented (Physiotherapy, Surgery, Combined Physiotherapy/Surgery, Medication/Orthotics, Education/Other). Video popularity was determined by the use of View Ratio (VR)(Erdem & Karaca, 2018; Rodriguez-Rodriguez, Blanco-Diaz, Lopez-Diaz, de la Fuente-Costa, Duenas, et al., 2021) (see Equation (1)):

(1) $$VR=\frac{View\;Count}{Days\;Online}$$

Equation (1). *View Ratio Equation*.

The DISCERN scale(Charnock; Charnock et al.) and the Global Quality Scale (GQS) (Cakmak & Mantoglu, 2021) were used to assess the reliability and educational quality of the 108 included videos. Video accuracy and reliability were evaluated using the benchmark criteria established by the Journal of American Medical Association (JAMA)(Silberg et al., 1997). Understandability and Actionability of patient education materials were evaluated with the Patient Education Materials Assessment Tool (PEMAT)(M. Agency for Healthcare Research and Quality, 2020). The overall quality of online medical videos was assessed with the recently developed Medical Quality Video Evaluation Tool (MQ-VET)(Guler & Aydın, 2022).

##### 2.2.1.1 DISCERN tool

In this study, a modified version of the original five-point DISCERN tool was used to assess the reliability and educational quality of the records. This validated and adapted tool comprises five distinct questions, assigning one point if the video satisfies the respective topic and zero points if it does not. The original validated questionnaire, named "Quality Criteria for Consumer Health Information," was initially developed by the "Public Health and Primary Care Division" of Oxford University (London) to evaluate the information quality pertaining to treatment choices for health issues. It was first published in 1999 (Charnock). DISCERN scores being within the range of 4–5 points are categorized as "Very High," scores between 3 and 4 are labeled as "High," those between 2 and 3 are considered "Average," scores from 1 to 2 are classified as "Low," and scores between 0 and 1 are denoted as "Very Low." Higher scores on the scale indicate greater levels of information quality(Charnock et al.).

##### 2.2.1.2 Global quality score (GQS) tool

This validated tool assesses the overall quality of online resources by evaluating five criteria in each video. Similar to the DISCERN Tool, each of the five identifiable criteria present in a video is assigned one point, with a maximum educational quality score of 5 (Kunze et al., 2019). This scale encompasses aspects such as the information quality and accessibility, the overall flow of information, and the usefulness of the content for users(Cakmak & Mantoglu, 2021).

##### 2.2.1.3 JAMA tool

The assessment of video accuracy and reliability was carried out using the benchmark 4-criteria assessment tool established and validated by the Journal of American Medical Association (JAMA): Authorship, Attribution, Disclosure and Currency(Silberg et al., 1997). The authorship criterion focuses on accurately stating the author's name and affiliation on the website. The attribution item measures the effectiveness of referencing the content provided on the website. The currency criterion evaluates whether the website includes the date of uploading and updating. Lastly, the disclosure category indicates whether the site owner has highlighted any potential conflicts of interest. Observers score each item on a scale of 1–4 points(Eysenbach et al., 2002; Salman & Bayar, 2021).

##### 2.2.1.4 Patient Education Materials Assessment Tool (PEMAT)

The Patient Education Materials Assessment Tool (PEMAT), a valid and reliable instrument to evaluate Understandability and Actionability of patient education materials, was developed and validated by the Agency for Healthcare Research and Quality (AHRQ)(M. Agency for Healthcare Research and Quality, 2020). Understandability refers to the ability of diverse individuals to comprehend and explain key messages. Actionability assesses whether varied health literacy levels and backgrounds can determine appropriate health actions based on the information. PEMAT has two parts: PEMAT-P for print materials like brochures and PEMAT-A/V for audiovisuals like videos, the latter being used here. Scores are calculated by summing points, and dividing by total possible points. Maximum is 1.0 for Understandability, 1.0 for Actionability, totaling 2.0 maximum. Developers established a cutoff of 0.7 for both Understandability and Actionability(Furukawa et al., 2022; Vishnevetsky et al., 2018).

##### 2.2.1.5 Medical Quality Video Evaluation Tool (MQ-VET)

The Medical Quality Video Evaluation Tool (MQ-VET) is a validated questionnaire evaluating online medical video quality from both health professionals and general public(Guler & Aydın, 2022). Research shows MQ-VET is a reliable and valid instrument expected to play a crucial role in standardizing evaluations of online medical videos. The final MQ-VET consists of four parts assessing different aspects, each with varying questions: Part 1 has 5 questions, Part 2 presents 4, Part 3 has 3, and Part 4 shows 3 questions, totaling 15 questions for the entire tool. All these questions are assessed on a Likert scale ranging from 1 to 5 points (where "Strongly Disagree" scores 1 point and "Strongly Agree" scores 5 points). So, the total score is the sum of the scores from all the questions, with a maximum score of 75 points.

#### 2.2.2 Statistical assessment

Following the assessments by the two examiners, the Intraclass Correlation Coefficient was calculated. After this, a dataset was constructed with the mean score resulting from the scores given by each examiner to each variable analyzed, thus building a single large dataset. Two statistical analyses were conducted on this dataset. The first one considered the analysis of the data from the perspective of descriptive statistics, as well as the exploration of Pearson Correlation Coefficients among the variables that comprise the dataset(Schober & Schwarte, 2018; Zou et al., 2003).

The second analysis was performed using Artificial Intelligence algorithms applied to high-dimensional problems, requiring Machine Learning tools. These tools belong to the realm of automatic learning and, more specifically, they lie in the Unsupervised Learning (UL) which is focused on the exploration of underlying structures or relationships among variables within a dataset. Once these patterns are identified, the features uncovered through unsupervised learning can often be integrated into supervised learning models(Johnson et al., 2018). Unlike supervised learning, UL autonomously groups objects by discovering inherent data patterns and structures(Roohi et al., 2020). Factor Analysis (FA) generates new variables based on the original variables set, revealing hidden causes, known as latent variables, that contribute to the variability observed in the dataset. Using these latent variables, it reduces the number of variables while retaining the maximum amount of information and establishing a relationship between the observable causes and the newly created variables, referred to as factors or components(Jolliffe, 2002; Qu et al., 2002).

The aim of these analyses was to reduce the number of dimensions of the dataset to ease the visualization of the information that it contains without any loss of it(Xia et al., 2022; Xiang et al., 2021). There are several methods for this purpose and one of the most known and most used of them is the Principal Component Analysis, among others. In this study, the clustering of the dataset has been made using three different AI techniques: Principal Component Analysis (PCA), t-distributed Stochastic Neighbor Embedding (t-SNE) and Uniform Manifold Approximation and Projection (UMAP)(McInnes et al., 2018a). HeatMaps(Wilkinson & Friendly, 2012) and Hierarchical Clustering Dendrogram will be used as another graphical confirmation of the clustering results (Öztürk & Çelik, 2022).

##### 2.2.2.1 Principal Component Analysis (PCA)

PCA is a data analysis technique that aims to extract essential information from a dataset comprising inter-correlated dependent variables(Lu et al., 2022). By employing an eigen-decomposition of positive semi-definite matrices and singular value decomposition (SVD) of rectangular matrices, PCA computes new orthogonal variables known as Principal Components. These components are linear combinations of the original variables and serve to represent the significant information in the data table(Wang et al., 2023), (Wang et al., 2021). One widely used method for rotation in PCA is Varimax, developed by Kaiser(Kaiser, 1958). Varimax rotation simplifies interpretation by promoting components with a small number of large loadings and a large number of zero or small loadings. This allows each original variable to be associated with one or a few components, and each component represents only a small subset of variables. Additionally, varimax facilitates interpretation through opposing positive loadings with negative loadings. Specifically, varimax seeks a linear combination of the original factors that maximizes the variance of squared loadings(Abdi & Williams, 2010).

PCA introduced a Measure of Sampling Adequacy (MSA) for factor analytic data matrices, adapted by Kaiser and Rice. The MSA calculates the squared elements of the matrix in relation to the squared original correlations(Kaiser, 1970). This measure, known as the Kaiser-Meyer-Olkin (KMO) index, is categorized as "Unacceptable" if below 0.50, "Miserable" between 0.50 and 0.60, "Mediocre" above 0.60 and below 0.70, "Middling" between 0.70 and 0.80, "Meritorious" between 0.80 and 0.90, and classified as "Marvelous" if above 0.90 (but less than 1.00) (Kaiser, 1982). The KMO test, which examines multivariate normality and sample adequacy, was utilized to assess construct validity and determine the suitability of the sample for factor analysis. Bartlett's Test of Sphericity(Bartlett, 1951) was employed to evaluate the sample's suitability for factor analysis and ensure the statistical power of the analysis conducted, as indicated by a combination of PCA analysis and the KMO index (Lastovicka & Jackson, 1992).

##### 2.2.2.2 t-distributed Stochastic Neighbor Embedding (t-SNE)

t-SNE is, contrary to PCA, a nonlinear dimensionality reduction technique that visualizes high-dimensional data in a lower 2 or 3 dimensional space. It represents each high-dimensional object as a 2D or 3D point, positioning similar objects close together and dissimilar objects far apart(Linderman & Steinerberger, 2019).

The t-SNE algorithm, developed by Laurens van der Maaten(Van Der Maaten & Hinton, 2008), has three main steps. First, it calculates the similarities between neighboring points in the initial high-dimensional space, using a Gaussian distribution centered on each point. These similarities are converted into observed conditional probabilities and the standard deviation is defined by a value called perplexity which corresponds to the number of neighbors around each point. The second step creates a smaller dimensional space and randomly places the points in it. Points similarities in the new space are calculated using a t-Student distribution instead of a Gaussian distribution, obtaining a list of probabilities. In the third step, the goal is to match the similarities between the original space and the lower-dimensional space. This is achieved by minimizing the Kullback-Leibler (KL)(Kullback & Leibler, 1951) measure, which compares the probability distributions of the two spaces. The algorithm uses gradient descent to find the best possible representation of the data points in the lower-dimensional space. In summary, t-SNE provides a way to visualize complex high-dimensional data by mapping it to a lower-dimensional space while preserving data point similarities(Cieslak et al., 2020; Zhou & Sharpee, 2022).

For the t-SNE calculation, the perplexity value is of very high importance. The perplexity is defined as follows in Equation (2):

(2) $$Perp\left(P_{i}\right)=2^{H\left(P_{i}\right)}$$

Equation (2). *Perplexity in t-SNE.*

where H(Pi) is the Shannon Entropy of Pi measured in bits (Shannon, 1948) (Equation (3)):

(3) $$H\left(P_{i}\right)=-\;\sum_{j}p_{i|j}\;.\;\log_{2}p_{j|i}$$

Equation (3). *Shannon Entropy of Pi in t-SNE.*

After calculating the Euclidean distances between each data point and all other points and converting these distances into conditional probabilities that represent the similarity between every two points, the conditional probability of point x_j_ to be next to point x_i_ is represented by a Gaussian probability distribution which is centered at x_i_ with a standard deviation of ***σ***_i_, as it is expressed in Equation (4):

(4) $$p_{\frac{j}{i}}=\frac{\exp\left({-∥x_{i}-x_{j}∥}^{2}/\;2\sigma_{i}^{2}\right)}{\sum_{k\neq i}\exp\left({-∥x_{i}-x_{k}∥}^{2}\;/\;2\sigma_{i}^{2}\right)}$$

Equation (4). *Conditional probability in t-SNE.*

With the conditional distributions, the joint probability distribution is build using Equation (5) that will be used in the low-dimensional space but, instead of using the Gaussian distribution, the t-distribution will be used because of its heavy tails property that makes moderate distances between points in the high-dimensional space to become extreme in the low-dimensional space(Equation (6)).

(5) $$p_{ij}=\frac{p_{j|i}+p_{i|j}\;}{2n}$$

Equation (5). *Joint probability distribution in t-SNE.*

(6) $$q_{ij}=\frac{{\left(1+{∥y_{i}-y_{j}∥}^{2}\right)}^{-1}}{\sum_{k\neq l\;}{\left({1+∥y_{k}-y_{l}∥}^{2}\right)}^{-1}}$$

Equation (6). *Joint probability distribution with t-distribution in t-SNE.*

##### 2.2.2.3 Uniform Manifold Approximation and Projection (UMAP)

UMAP is a novel dimensionality reduction technique using Riemannian geometry and algebraic topology, developed in 2018 by McInnes(McInnes et al., 2018b). It provides a practical and scalable algorithm applicable to large datasets. UMAP competes with t-SNE in terms of visualization quality while better preserving the global structure and demonstrating superior runtime performance. UMAP has no computational constraints on embedding dimension, making it suitable for general machine learning dimensionality reduction(Becht et al., 2018; McInnes et al., 2018c). UMAP assumes that the data has a uniform distribution on a Riemannian manifold, with locally constant metric and local connectivity. It leverages these assumptions to model the manifold with a fuzzy topological structure and searches for a low-dimensional projection that closely preserves this structure(Demidova & Gorchakov, 2022).

In summary, UMAP, like t-SNE(Kobak & Linderman, 2021; Roca et al., 2023), is a powerful nonlinear dimensionality reduction technique that finds low-dimensional embeddings of structured data by incorporating Riemannian geometry and algebraic topology. It provides competitive visualization quality and broad applicability for machine learning tasks(Sainburg et al., 2020; Zolotenkova et al., 2022).

As with previous techniques, the dataset samples were represented in a new coordinate system with the UMAP algorithms but, instead of Perplexity in t-SNE, UMAP uses the number of k nearest neighbors as follows (Equation (7)):

(7) $$k=2^{\sum_{i}p_{ij}}$$

Equation (7). *Number of k-nearest neighbors in UMAP.*

where the symmetrization of the high dimensional probability is different to t-SNE, as indicated in Equation (8):

(8) $$p_{ij}=p_{i|j}+p_{j|i}-\;p_{i|j\;}p_{j|i}$$

Equation (8). *Crossed probability in UMAP.*

and Equation (9):

(9) $$p_{i|j}=e^{-\;\frac{d\left(x_{i},{\;x}_{j}\right)\;-\;\rho_{i}}{\sigma_{i}}}$$

Equation (9). *Exponential probability distribution in high dimensions in UMAP.*

After this, UMAP calculates the distance probabilities in low dimensions with a modified t-Student distribution with no applied normalization as indicated in Equation (10).

(10) $$q_{ij}={{(1+a\left(\;y_{i}-\;y_{j\;}\right)}^{2b})}^{-1}$$

Equation (10). *Distance probability distribution in low dimensions in UMAP.*

Finally, instead the KL-divergence used in t-SNE, UMAP uses binary cross-entropy, as indicated in Equation (11):

(11) $$CE\left(X,Y\right)=\sum_{i}\sum_{j}\left[p_{ij}\left(X\right)\log\left(\frac{p_{ij}\left(X\right)}{q_{ij}\left(X\right)}\right)+\left(1-\;p_{ij}\left(X\right)\right)\log\left(\frac{{1-p}_{ij}\left(X\right)}{1-q_{ij}\left(X\right)}\right)\right]\;$$

Equation (11). *Binary cross-entropy in UMAP.*

##### 2.2.2.4 Heat map and Hierarchical Clustering Dendrogram

A heat map graphically represents data where individual values contained in a matrix are represented using colors. Clustering análisis refers to the process of grouping a set of samples together based on the similarity of their internal expression patterns. This analysis has two primary applications. Firstly, it serves as a quality control measure by identifying outliers within the dataset and, secondly, it enables the classification of sample subtypes based on their expression patterns(Zhao et al., 2014).

The cluster heat map is a smart visualization technique that arranges a data matrix in a rectangular format, with additional cluster trees attached to its edges. This arrangement allows for a compact display area and facilitates comparisons between rows (variables), columns (samples), and the overall cluster structures. The popularity of heat map cluster analysis stems from its ability to present large amounts of information (even thousands of rows/columns) in a visually concise manner, revealing coherent patterns in the data (Nthai et al., 2022; Wilkinson & Friendly, 2012). The cluster heat map serves as an innovative display that simultaneously showcases the hierarchical cluster structures of both rows and columns in the data matrix. It employs a rectangular tiling where each tile is shaded on a color scale, representing the corresponding value in the data matrix. The ordering of rows and columns in the tiling ensures that similar ones are placed close together, which is one of the main advantages of this technique. The hierarchical cluster trees are positioned along the vertical and horizontal margins of the tiling. This cluster heat map amalgamates various graphic displays developed by statisticians over the course of many years with different fields of application.

In various health-related domains, including biology and genomics, clustered heat maps are widely utilized as the preferred graphical tool for visualizing and interpreting large datasets. They offer an effective mean of representing complex information in a visually intuitive manner. The creation of a heat map needs the expertise of a biostatistician or bioinformatics analyst who possesses proficiency in advanced statistical techniques and is skilled in programming languages that enable the basic steps for its construction: application of hierarchical clustering methods, association of covariate (classification) data, and production of the heat map visualization(Broom et al., 2020). Indeed, cluster analysis constitutes an ideal tool for detecting outlier samples in large dataset studies(Hai et al., 2006). An illustrative example is the breast cancer study conducted as part of The Cancer Genome Atlas (TCGA) project(Koboldt et al., 2012), in which clustering techniques were employed to uncover distinct subtypes of samples based on their variables’ patterns. This application of cluster analysis facilitated the identification and characterization of different breast cancer subtypes within the dataset.

Hierarchical cluster analysis, a modern multivariate statistical method, was utilized to identify the clustering tendency of variables. The relationships between variables were visually represented as a dendrogram, which reveals connections among similar objects. The dendrogram was constructed using common linkage (between groups) and Ward's hierarchical clustering model, chosen to minimize variability within clusters and maximize variability between them. This approach allows controlling associations between variables by collectively examining multiple variables to investigate interactive outcomes and determine the links between variables or specific conditions under which associations occur. Hierarchical cluster analysis, through its incorporation of multiple variables, furnishes a more comprehensive and authentic portrayal when compared to the examination of a single variable. Additionally, it presents a robust significance testing capability, diverging notably from univariate methodologies. Consequently, it produces more robust outcomes compared to univariate techniques(Çelik, 2017; Öztürk & Çelik, 2022). The dendrogram generated by hierarchical clustering is a cluster tree that depicts data organization according to their clustering proximity. During this procedure, clusters are created by linking two or more subsequent clusters, comprising similar data for each tree cluster. The dendrogram provides a hierarchical structure that visually demonstrates the relationships and similarities between different data groups(Langfelder et al., 2008; Nthai et al., 2022).

All analyses were conducted using SPSS version 26 (IBM, Armonk, NY, USA) and XLSTAT (Addinsoft, Paris, France), which is a statistical software that can be employed to perform multivariate analysis of complex data sets and provides a comprehensive suite of tools for Data Analysis, Machine Learning, and Statistical Solutions for MS Excel (Microsoft Corporation, Redmond, WA, US), which was also used. The software used for performing the t-SNE and UMAP analysis was Qlucore Omics Explorer (Qlucore, Lund, Sweden) which is a data analysis and data mining software tool built on state-of-the-art mathematical and statistical methods that combines speed and advanced analytics for interactive exploration and instant visualization of high-dimensional data. Preliminary analyses were implemented to avoid violation of the assumptions of normality, linearity, and homoscedasticity. To evaluate the concordance among examiners, an Interclass Correlation Coefficient (ICC) analysis was performed, employing the mean rating (k = 2), a consistency approach, the two-way random model, and Pearson's Correlation method. It was established the 95% confidence intervals (CIs) during the analysis. The level of significance was predetermined at p < 0.05, with ICC values categorized as follows: values below 0.5 denoting "Poor" reliability, values between 0.5 and 0.75 indicating "Moderate" reliability, values spanning from 0.75 to 0.90 signifying "Good" reliability, and values exceeding 0.90 representing "Excellent" reliability (Langfelder et al., 2008).

## 3 Results

### 3.1 Inter-reviewers concordance

The inter-rater agreement for this study yielded a score of 0.9465.

### 3.2 Descriptive statistics

For each video, descriptive statistics were computed individually to determine encompassing metrics such as the mean, standard deviation (SD), median, minimum and maximum values. The same calculations were performed for the quality scales. This information is shown in Table 1.

**Table 1:** Descriptive statistics of the variables recorded for the study.

	Mean	SD	Median	Min	Max
Days online	1921.57	1276.95	1614.50	81.00	5150.00
Duration (hh:mm:ss)	0:15:08	0:19:02	0:06:22	0:00:23	1:08:10
Views (n)	79,334.47	582,735.22	3376.00	40.00	6,032,889.00
Likes (n)	690.00	5504.28	18.50	0.00	57,174.00
Comments (n)	51.83	386.74	0.00	0.00	4018.00
Subscribers (n)	129,693.65	590,655.89	4570.00	0.00	5,870,000.00
View ratio (n)	49.63	341.47	2.50	0.00	3447.00
DISCERN	3.37	0.92	3.00	1.00	5.00
GQS	3.22	0.89	3.00	1.00	5.00
JAMA	2.48	0.72	2.00	1.00	4.00
PEMAT	1.35	0.29	1.38	0.65	1.92
MQ-VET	51.50	9.35	45.94	0.00	75.00

The information regarding the scores in DISCERN, GQS, JAMA, PEMAT and MQ-VET tools according to the Origin of the video, the Author (or uploader), the Gender and the Cancer Type, expressed in Mean and SD for each of them can be seen in Table 2.

**Table 2:** Mean and SD values of the scales according to Origin, Author, Gender and Cancer Type.

	DISCERN		GQS		JAMA		PEMAT		MQ-VET	
	Mean	SD	Mean	SD	Mean	SD	Mean	SD	Mean	SD
ORIGIN										
1. Africa	0.00	0.00	0.00	0.00	0.00	0.00	0.00	0.00	0.00	0.00
2. America	3.41	0.90	3.27	0.90	2.52	0.68	1.35	0.29	52.50	9.15
3. Asia	3.00	0.00	2.5	0.70	1.50	0.70	1.14	0.15	42.50	3.50
4. Australia	3.27	1.00	3.18	0.98	2.18	0.75	1.32	0.27	48.60	8.35
5. Europe	3.18	1.07	3.09	0.83	2.63	0.80	1.34	0.33	51.50	11.45
AUTHOR/UPLOADER										
1. Academic Institution	3.92	0.86	3.84	0.80	2.92	0.64	1.49	0.22	55.50	4.90
2. Media	3.25	0.95	3.5	1.29	2.50	0.57	1.50	0.35	53.50	11.05
3. Health institution	3.40	0.98	3.35	0.91	2.56	0.64	1.37	0.27	53.00	8.95
4. NGOs	3.28	0.48	3.14	0.69	2.28	0.95	1.25	0.26	48.55	10.65
5. Health Individuals	3.47	0.76	3.15	0.78	2.42	0.75	1.33	0.28	51.00	10.65
6. Non-Health individuals	2.11	0.60	2.11	0.33	1.88	0.33	1.09	0.35	45.55	4.60
GENDER										
1. Female	3.45	0.82	3.45	0.52	2.45	0.52	1.41	0.31	52.50	10.05
2. Male	3.36	0.93	3.20	0.93	2.48	0.73	1.33	0.29	51.50	9.30
CANCER TYPE										
1. Prostate	3.32	0.93	3.21	0.93	2.47	0.71	1.32	0.28	51.50	9.20
2. Uterus	4.50	0.70	4.00	0.00	3.50	0.70	1.59	0.10	62.50	3.50
3. Bladder	3.25	0.70	2.87	0.64	2.12	0.64	1.39	0.33	48.75	10.25
4. Colorrectal	0.00	0.00	0.00	0.00	0.00	0.00	0.00	0.00	0.00	0.00
5. Other	4.00	0.70	3.80	0.44	2.80	0.44	1.56	0.33	58.00	9.05
TOTAL	3.37	0.92	3.23	0.90	2.48	0.71	1.34	0.29	51.50	9.35

Considering the source of production (author or uploader of the videos), 35.18% of them were uploaded by Health Individuals, followed by Health Institutions (34.25%), Academic Institutions (12.03%), Non-Health Individuals (8.33%), NGOs (6.48%) and, finally, Media (3.70%). These results can be found in Table 3.

**Table 3:** Percentages of videos by Author.

AUTHOR/UPLOADER	%
1. Academic Institution	12.03%
2. Media	3.70%
3. Health institution	34.25%
4. NGOs	6.48%
5. Health Individuals	35.18%
6. Non-Health individuals	8.33%

Regarding the patient gender to which the video is oriented, 89.81% of the videos are intended for male cancer patients and 10.19% for female patients (Table 4). Among the female-tailored videos, the majority were focused on uterine cancer (4.63%), followed by other cancer types (4.63%), with a smaller percentage of bladder cancer (0.93%).

**Table 4:** Percentages of videos by Gender and Cancer Type.

Gender and Cancer Type	%
1. Female	10,19%
1. Prostate	0,00%
2. Uterus	4,63%
3. Bladder	0,93%
4. Colorrectal	0,00%
5. Other	4,63%
2. Male	89,81%
1. Prostate	82,41%
2. Uterus	0,00%
3. Bladder	7,41%
4. Colorrectal	0,00%
5. Other	0,00%

No videos addressing colorectal cancer were found for females. Conversely, among male-oriented videos, prostate cancer dominated with the highest percentage, accounting for 82.41% of the content. Bladder cancer constituted the second most prevalent topic at 7.41%, whereas there were no videos specifically addressing colorectal cancer or other types of cancer for male patients.

### 3.3 Correlation within quality scales

The Pearson Correlation Coefficients and the 95% Confidence Intervals limits can be seen in Table 5 for the quality scales in relation to each other. All of them present a high-statistically significant correlations (p < 00.01).

**Table 5:** Pearson correlation coefficients and confidence intervals of quality scales.

	Pearson Correlation	95% Confidence Intervals (bilateral)^a^	
		Inferior	Superior
**DISCERN - GQS**	0.926^b^	0.894	0.949
**DISCERN - JAMA**	0.772^b^	0.682	0.838
**DISCERN - PEMAT**	0.747^b^	0.650	0.820
**DISCERN - MQVET**	0.739^b^	0.640	0.814
**GQS - JAMA**	0.812^b^	0.737	0.868
**GQS - PEMAT**	0.791^b^	0.708	0.853
**GQS - MQVET**	0.803^b^	0.724	0.861
**JAMA - PEMAT**	0.697^b^	0.585	0.783
**JAMA - MQVET**	0.812^b^	0.736	0.867
**PEMAT - MQVET**	0.778^b^	0.690	0.843

a The estimation is based on the Fisher's transformation of R to Z.

b The correlation is significant at .01 level (bilateral).

Furthermore, statistically significant two-by-two correlations were observed between the DISCERN, GQS, JAMA, PEMAT and MQVET scales (all *p*-values <0.01), as delineated in Table 5. When evaluating educational quality using these scales, it is notable that the average score achieved by the videos was “High” per the DISCERN scoring system. DISCERN scores of 4–5 points are categorized as “Very High”, 3–4 as “High”, 2–3 as “Average”, 1–2 as “Low”, and 0–1 as “Very Low”. Higher scores indicate greater information quality(Charnock). Normalizing all scales between 0 and 5 (Table 6) revealed overall average dataset scores within the 3–4 range across scales, with minimal differences. This denotes that video quality can be considered “High” according to these scales. This finding bolsters the statistical analysis demonstrating significant correlations between the five applied scales.

**Table 6:** Normalized scores of the dataset in the questionnaires.

Scale	Score achieved	Maximum possible score	Normalized score (over 5.00)
DISCERN	3.37	5.00	3.37
GQS	3.23	5.00	3.23
JAMA	2.48	4.00	3.10
PEMAT	1.35	2.00	3.37
MQ-VET	51.90	75.00	3.46

### 3.4 Dimensionality reduction

#### 3.4.1 Principal Component Analysis (PCA)

**Fig. 2:**
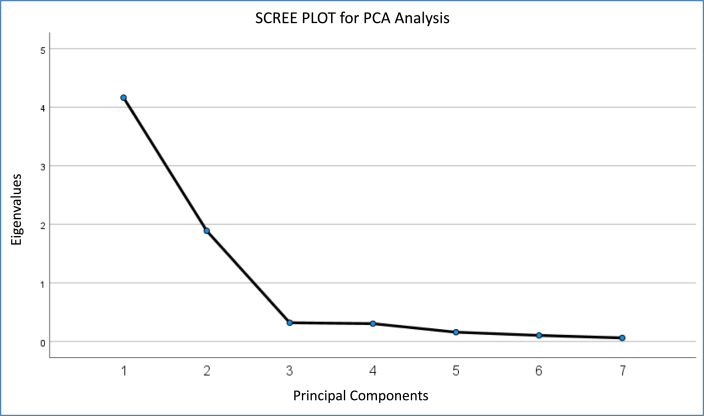
Scree plot for PCA analysis.

**Fig. 3:**
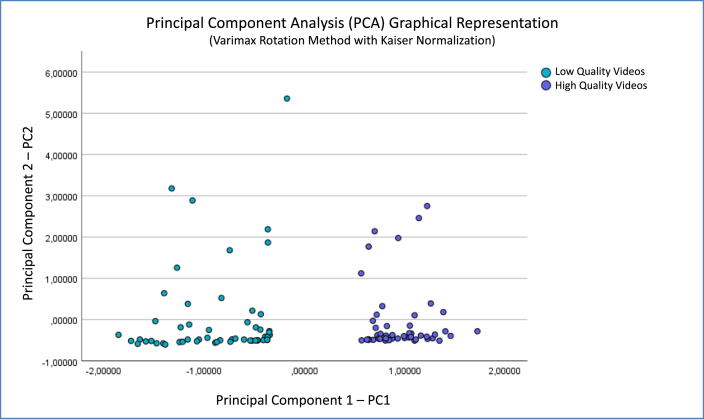
Principal Component Analysis (PCA) graphical representation of the sample.

In this PCA analysis, the KMO indicator for sample adequacy exhibited a score of 0.879 and the BST displayed a high-significance value (χ^2^ = 717.500; p < 0.001). The new reference system is established on the computed PCs, which are obtained as a linear algebraic combination of the original variables in the dataset. The total explained variance is 86.497% if the first two PCs are selected, as the Scree-Plot recommends (Fig. 2), which means that a significant amount of information from the original dataset is captured in the dimensional reduction process. Fig. 3 displays the distribution of the samples in the new orthogonal coordinated axes representation system.

#### 3.4.2 t-distributed Stochastic Neighbor Embedding (t-SNE)

**Fig. 4:**
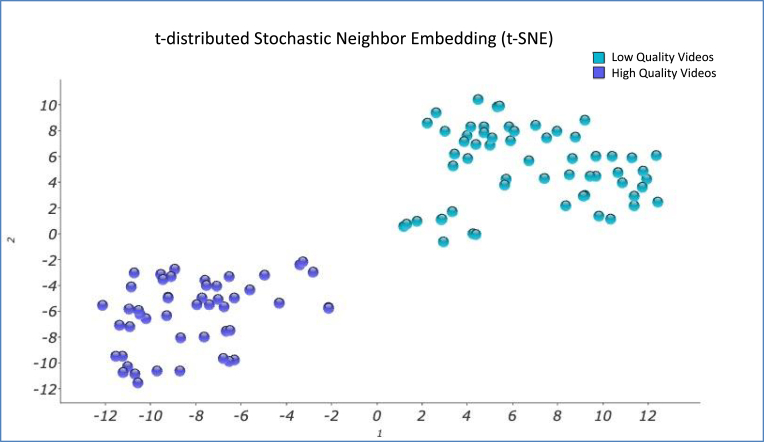
t-SNE graphical representation of the sample.

After calculating the probabilities for each pair of points, the Perplexity value can be interpreted as a smooth measure of the effective number of neighbors, has been set for this analysis to Perp = 10, which is a standard value in the typical range recommended for this sample size (Van Der Maaten & Hinton, 2008). The samples distribution of this study, after the t-SNE calculation, can be seen in Fig. 4.

#### 3.4.3 Uniform Manifold Approximation and Projection (UMAP)

**Fig. 5:**
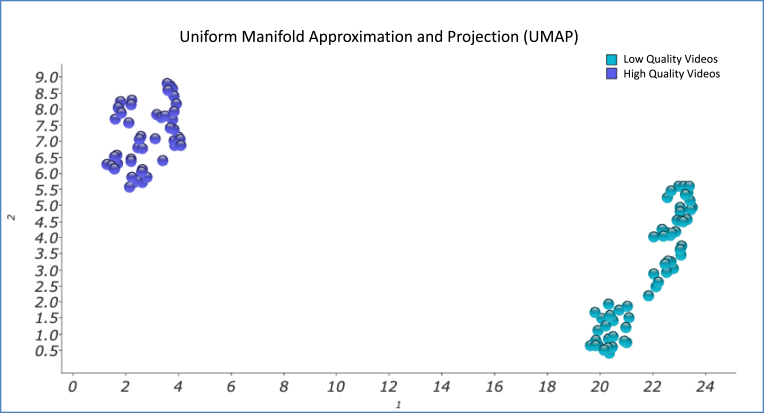
UMAP graphical representation of the sample.

Similarly to t-SNE, the value of the k-nearest neighbors was settled at k = 10. The samples distribution after the UMAP calculation can be seen in Fig. 5.

#### 3.4.4 Heat map and Hierarchical Clustering Dendrogram

**Fig. 6:**
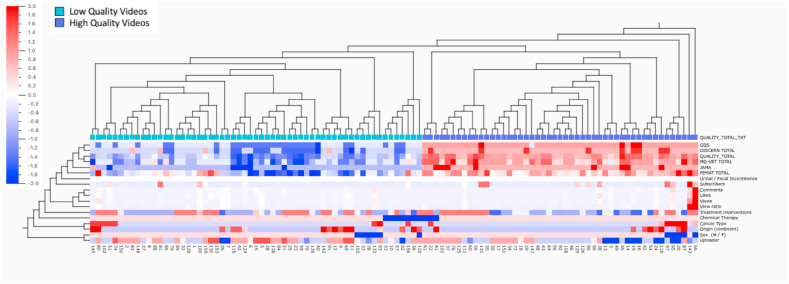
Heat map and hierarchical clustering dendrogram.

Fig. 6 delineates the construction and graphical representation of the Heat Map and Dendrogram, exhibiting clear differentiations in color density and hierarchical sample grouping within the dataset. The samples constitute the columns and the analyzed study variables comprise the rows. The first colored row depicts the primary sample clustering classification based on similarity, considering the multidimensional contributions of all variables to each dataset sample.

## 4 Discussion

This study aimed to gain greater understanding of the health information independently accessed by patients on a popular online media-sharing platform, specifically in Youtube. With the widespread adoption of new technologies and vast amount of available content, the Internet has become a major source of health information for healthcare professionals, patients and general public alike. However, this accessibility poses certain risks, as online information is not subjected to any form of scrutiny. Consequently, misleading or even harmful messages can be disseminated to users and viewers. To enhance healthcare processes, rigorous education and dissemination, preferably carried out by healthcare professionals, should be prioritized. Among the analyzed videos, the majority were produced by Health Individuals, Healthcare Institutions and Academic Institutions (35.18%, 34.25% and 12.03%, respectively) providing a degree of reliability, as they ranked highly on quality scales. Indeed, these three author types, normally used by other authors (Rodriguez-Rodriguez et al., 2024), obtained the highest quality scores across the considered scales, consistently led by Academic Institutions. Non-Health Individuals uploaded a substantial number of videos (8.33%) but these videos obtained the lowest scores in all the quality scales without exception.

In our study, the analyzed videos had an average duration of 15:08 min, which is aligned with the average lengths reported in previous studies that presented a mean length of 14:42 min (Rodriguez-Rodriguez, Blanco-Diaz, Lopez-Diaz, de la Fuente-Costa, Sousa-Fraguas, et al., 2021), regarding the quality of videos presenting pelvic floor exercises after prostatectomy surgery. Interestingly, it was not observed a statistically significant correlation between video length and their quality or popularity.

The Intraclass Correlation Coefficient (ICC) is a commonly employed measure to assess test-retest, intra-rater, and inter-rater reliability. Utilizing the 95% confidence interval of the ICC estimation(Koo and Li), the inter-reviewer agreement in this study was determined to be 0.9465, indicating an Excellent level of concordance between the two examiners.

In this health research study, Pearson correlation analysis was conducted between the utilized quality scales (DISCERN, GQS, JAMA, PEMAT and MQ-VET). As delineated above in Table 5, Table 6, the statistical analysis demonstrated strong, highly significant positive correlations (with p-values <0.01) between these scales, indicating a high degree of coherence and convergence. Thus, the comprehensive statistical correlation analysis reveals significant interrelationships and robust agreement between these scales for evaluating the quality of the information assessed.

From the perspective of the second part of the statistical analysis based on Artificial Intelligence algorithms, where the aforementioned Machine Learning techniques framed in the Unsupervised Learning part are applied, it can be seen that all of them show graphical results that support the auto-clustering of the dataset samples.

Initially, the scree plot from the PCA analysis indicated that the first two principal components (PCs), with eigenvalues greater than 1, could provide graphical representation explaining 86.497% of the complete dataset information. This denotes that these two PCs, comprising linear combinations of all study variables, could analyze and represent the information with sufficient certainty, without omitting important information. By plotting the dataset sample coordinates on the new orthogonal axis system constituted by the PCs themselves, two clearly differentiated sample groups or clusters were obtained. Assigning the color green to samples with scores below the dataset average, and blue to those above average, revealed no mixing of colored points. Rather, each color clustered on distinct sides, demonstrating automatic self-clustering of points without human intervention that could introduce bias.

Performing the same procedure but with the t-SNE tool described above, it is observed that the differentiation of the points clusters is even clearer, due to the fact that the clusters are more distant from each other and, therefore, more differentiated among them. This may also be a consequence of the characteristics of the calculation method itself, which is non-linear and considers probabilistic aspects of the sample densities in the graph space.

However, if the same procedure is performed using the UMAP algorithms, the clustering is even clearer than in the two previous cases, since there is a greater inter-group distance and a smaller intra-group distance, giving rise to a very significant visualization of the automatic behavior of the dataset samples.

The dendogram analysis shows the groupings of the samples according to different criteria and the values obtained for different variables. Thus, as can be seen in the vertical self-classification lines of the dendogram (not the heatmap perspective but the dendogram view) there are two clearly differentiated groups of high and low quality. The determination of the main trends of the video values is done by means of the mean values and standard deviations according to the self-classification and clustering derived from the previous mentioned analysis.

The interpretation of the results of these calculation methods based on Artificial Intelligence tools reinforces each other without any contradiction in any of the analyzed cases. These calculation methods allow a graphical representation of the behavior of the samples without human intervention and show a clear differentiation of the point clouds through the two clusters that are formed automatically, so they are tools that can also be applied in this kind of analysis as their use and effectiveness has been demonstrated in many studies in different areas of health, especially in those oriented to genomics, biomolecule analysis, DNA studies, metabolomics, microbiome analysis, epidemiology, brain imaging, biomarker discovery, etc.

Thus, it can be seen in these graphical representations of the different methods used for this analysis that the auto-clustering of the videos without human intervention in this process is clear, so that all the variables are considered in this analysis and no relevant part of the information is left unconsidered in these five analyses.

Healthcare institutions, Academic Institutions and Healthcare Professionals adhere more strictly to accurate terminology for these types of activities compared to other sources that simply refer to them without specifying the term. The use of appropriate terminology is crucial due to the growing cultural awareness within society. People desire to perceive themselves as active decision-makers rather than mere patients, and as a result, they expect a level of self-sufficiency in addressing their needs, which includes utilizing online resources for self-guided training. However, in order to facilitate effective learning, it is essential to employ suitable terminology for each specific situation(Boixareu, 2019; Rodriguez-Rodriguez, Blanco-Diaz, Lopez-Diaz, de la Fuente-Costa, Sousa-Fraguas, et al., 2021).

To address the specific utility of the PCA, t-SNE and UMAP artificial intelligence tools in the analysis of clinical videos, these methods have been instrumental in enabling a high-level view of complex, multidimensional data. The PCA offered a reduction in dimensionality, presenting a clear visualization of the dataset and highlighting the intrinsic structure within the data, which can be especially challenging to discern in the multifaceted context of clinical video content. The t-SNE and UMAP tools further facilitated this process by providing a refined clustering of the data, allowing for the elucidation of patterns that might otherwise be obscured due to the non-linear nature of health information. These AI tools have proven invaluable not only in the segmentation of data for a more granular understanding of content quality but also in mitigating the risk of human bias in the classification and analysis of such data. The application of PCA, t-SNE and UMAP in this study represents an innovative approach to the evaluation of educational content, offering a precedent for future studies in health informatics to harness the power of AI for enhanced data analysis and interpretation.

## 5 Limitations

In alignment with principles of simulating real user experiences, the search was conducted under incognito mode to avoid potential influences of browsing history and geographical location. While acknowledging that YouTube content continuously evolves, this analysis provides an accurate representation of the videos' quality at a defined moment(Rodriguez-Rodriguez, Blanco-Diaz, Lopez-Diaz, de la Fuente-Costa, Sousa-Fraguas, et al., 2021).

Only videos directly accessed on YouTube upon searching for “Incontinence after cancer surgery” were included in this study. External links from other medical-related websites were deliberately not considered in the analyses.

Given YouTube's dynamic landscape with ongoing uploading, viewing, and rating of new videos, it is imperative to note that this search was restricted to the first 160 videos. This approach aimed to mirror typical behavior of average users/patients, as previous and extensive research indicates most individuals do not extend searches beyond the initial 50 results(Steinberg et al., 2010).

## 6 Conclusions

Our research has comprehensively evaluated the quality and accuracy of health information available on YouTube concerning incontinence after pelvic cancer surgery. The primary aim of assessing the content's reliability was achieved using validated quality assessment tools (DISCERN, GQS, JAMA, PEMAT, and MQ-VET), which demonstrated high statistical correlations with one another, ensuring the scales' consistency in measuring information quality. The application of AI techniques such as PCA, t-SNE, and UMAP has proven effective for data visualization and pattern recognition, underpinning the clustering of data and enhancing the objectivity of the analysis by minimizing human bias.

In alignment with our secondary objective, this study's findings are instrumental for guiding healthcare providers and patients towards reliable sources of health information. Videos from Academic and Health Institutions and Health Professionals were rated highest in quality, suggesting their potential as trustworthy educational resources. In contrast, content from Non-Health Individuals was found to be less reliable. Future directives should emphasize the careful selection of information sources by patients, prioritizing content from established healthcare entities.

In conclusion, our study has determined that the overall quality of YouTube videos on the subject of 'Incontinence after Cancer Surgery' is high, as evidenced by the strong statistical correlations among the five quality assessment scales used: DISCERN, GQS, JAMA, PEMAT, and MQ-VET. Moreover, the integration of AI-based tools like PCA, t-SNE, and UMAP for clustering large health datasets demonstrates their value in enhancing data visualization and pattern recognition, enabling more effective analysis of complex healthcare information. Recognizing the lack of a specific indicator to discern high-quality videos, it is suggested that patients use the source of production as a guiding criterion, with content from Academic Institutions, Health Institutions, and Health Professionals being the most reliable. To avoid biased information, videos containing commercials should be approached with caution. Content produced by Non-Health individuals, which scored the lowest across all quality scales, should be viewed critically or avoided to ensure the integrity of health information consumption.

## Future research

The involvement of Academic and Health Institutions, along with Health Professionals, Government Health Authorities, and legislators, is crucial in formulating policies that enhance the quality of information available on the internet, leading to a positive influence on the health behaviors of the population. According to this, it is essential, for future research, to create and identify tools that assist patients in differentiating the highest quality health-related videos more easily. In future studies, the emphasis should be on establishing criteria for the effective use of YouTube as a supportive health information tool, highlighting the complementarity to, rather than substitution for, professional medical advice. This will be key to empowering users in discerning the quality of health content online.

## Funding

This research received no external funding.

## Informed consent statement

Not applicable.

## Declaration of generative AI in scientific writing

During the preparation of this work the authors used no AI tool/service for scientific writing in any of the parts of the manuscript. The authors take full responsibility for the content of the publication.

## CRediT authorship contribution statement

**Alvaro Manuel Rodriguez-Rodriguez:** Writing – original draft, Visualization, Software, Methodology, Investigation, Formal analysis, Conceptualization. **Marta De la Fuente-Costa:** Software, Data curation. **Mario Escalera-de la Riva:** Resources, Data curation. **Borja Perez-Dominguez:** Resources, Data curation. **Gustavo Paseiro-Ares:** Validation, Investigation. **Jose Casaña:** Writing – review & editing, Validation, Supervision, Methodology, Investigation. **Maria Blanco-Diaz:** Writing – review & editing, Writing – original draft, Visualization, Validation, Supervision, Resources, Methodology, Investigation, Conceptualization.

## Declaration of competing interest

The authors declare no conflict of interest.

## Data Availability

Data will be made available on request.
